# 841. An Evaluation of Outcomes and Hospital Readmissions Among Individuals with Candidemia in Connecticut (2019-2020)

**DOI:** 10.1093/ofid/ofad500.886

**Published:** 2023-11-27

**Authors:** Elizabeth Suschana, David Banach, James Meek, Paula Clogher, Maria Correa

**Affiliations:** UConn School of Medicine, Farmington, Connecticut; UConn Health, Farmington, Connecticut; Connecticut Emerging Infections Program, Yale School of Public Health, New Haven, Connecticut; Connecticut Emerging Infections Program, Yale School of Public Health, New Haven, Connecticut; Connecticut Emerging Infections Program, Yale School of Public Health, New Haven, Connecticut

## Abstract

**Background:**

In addition to significant morbidity and mortality, sepsis is a leading cause of hospital readmission. Candidemia, a leading cause of sepsis, has a particularly high morbidity and mortality rate. Previous studies defined risk factors for poor outcomes and readmission after general sepsis. However, specific risk factors for readmission after candidemia are unknown.

**Methods:**

The study is retrospective cohort study of adults in Connecticut with an episode of candidemia between 2019-2022 using statewide surveillance data. Individuals were classified based on primary outcome (died versus survived) at incident admission. Candidemia survivors were further classified by readmission status. A descriptive analysis of demographics, healthcare related factors, and readmission characteristics was performed in univariate and multivariate analyses to identify factors associated with mortality and readmission after an episode of candidemia.

**Results:**

Among 347 candidemia cases, 226 (65.1%) survived the incident episode. Those who survived were more likely to be younger (age > 65 = 70% vs age < 65 = 59.2%; p = 0.036), have community-onset infections (84.4% community-onset vs 47.2% hospital-onset; p < 0.001), and have an infection from a non-*Candida albicans* species (56.5% with *C. albicans* vs 70.8% with non-C. *albicans*; p = 0.006). In multivariate analysis, mortality was associated with hospital-onset infection [OR = 6.866, CI = 4.009 – 11.760]. Among survivors of the incident episode (n = 128), 56% were readmitted within 180 days post- incident episode. Of those readmitted, 13 (10.1%) were readmitted for a new incident candidemia episode. Readmission was associated with hospital-onset infection (OR=1.791, CI = 1.008 – 3.184).

**Table 1.**
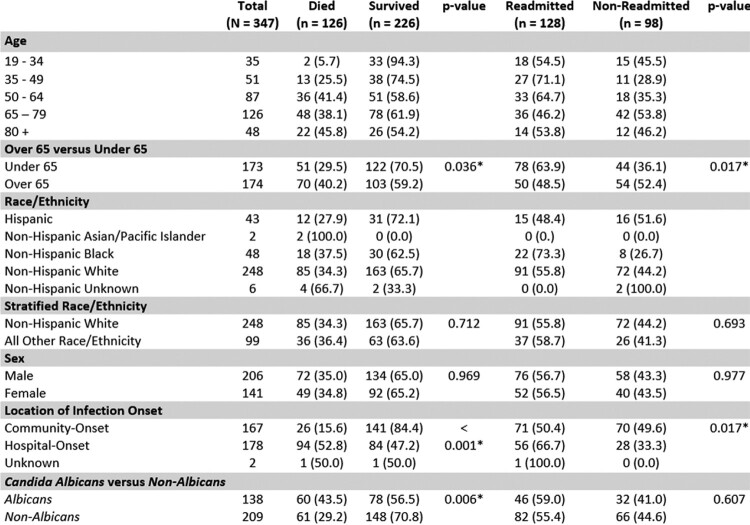
Demographic and clinical factors of incident case outcome and readmissions, 2019-2020 (Abbreviated).

* Indicates a significant p-value (p < 0.05)

**Table 2.**
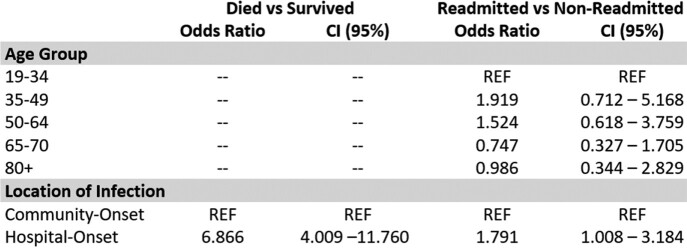
Multivariate analysis for age and class of infection by incident case outcome and readmission status

**Conclusions:**

Individuals with candidemia have a high risk of mortality and readmission. Individuals with hospital-onset infection are at particularly high risk of mortality and subsequent readmission. Efforts to prevent hospital-onset candidemia are critical. Additional measures focused on individuals with risk factors for readmission are needed to reduce overall morbidity and mortality associated with candidemia.

**Disclosures:**

**All Authors**: No reported disclosures

